# The Scatchard Equation for a Multivalent Ligand: A Much Needed but Neglected Expression Required for Valid Interpretation of Biphasic Bioassays

**DOI:** 10.1007/s10930-026-10334-8

**Published:** 2026-05-11

**Authors:** Donald J. Winzor

**Affiliations:** School of Chemistry and Molecular Biosciences, University of Queensland, Brisbane, QLD 4072 Australia

**Keywords:** Protein–ligand interactions, Ligand multivalence, Quantitative affinity chromatography, Radioimmunoassays, Erythrocyte ghosts, Muscle myofibrils

## Abstract

Attention is drawn to the availability of a version of the Scatchard expression that accommodates multivalence of the ligand. Its lack of application over the past four decades by biological researchers is surprising in view of the popularity of its univalent counterpart. This summary of its derivation and limited application presents published examples of situations where the curvilinearity of conventional Scatchard plots has been misinterpreted as signifying heterogeneity or negative cooperativity of binding sites on erythrocyte ghosts instead of tetravalence of glycolytic enzymes in their interaction with band 3 protein. Also presented is the rectangular hyperbolic relationship of which the generalized Scatchard expression is the linear transform.

## Introduction

From the middle of last Century, the Scatchard rearrangement [1] of the basic binding equation [2] found widespread application for the quantitative characterization ligand interactions with proteins. However, little attention was given to the fact that valid use of the Scatchard plot is conditional upon univalence of the ligand, an assumption inherent in the Klotz [2] and hence Scatchard [1] expressions. This limitation was rendered even more problematic by the advent of quantitative affinity chromatography [3, 4], where the protein is the species undergoing partition between free and bound states. Assumed univalence of a protein exhibiting quaternary structure clearly became a questionable approximation.

The solution to this dilemma came in a quantitative affinity study of the interaction between aldolase and cellulose phosphate [5], where a shift from the Klotz [1] treatment of site equivalence and independence to the corresponding concept of reacted-site probability [6] led to a quantitative expression for the interaction of a multivalent ligand with immobilized affinity sites. Facilitation of its use by means of a linear rearrangement [7] quickly led to the realization [8] that the linear transform was the multivalent counterpart of the Scatchard equation. The stage was thus set for extension of the Scatchard analysis to a much wider range of protein interactions.

Unfortunately, the significance of this more general counterpart of the Scatchard analysis has been overlooked by the biological research community in that its application has been limited to studies by researchers involved in its development. The current review highlights this oversight by drawing attention to situations where curvature of the standard Scatchard plot merely reflected nonconformity with the assumption of ligand univalence that is a condition for its validity. Also presented are results from studies of biologically relevant biphasic interactions to revitalize interest in the importance of the breakthrough afforded by the availability of a multivalent counterpart of the Scatchard plot, an important but seemingly neglected expression.

## The Binding of a Univalent Ligand to a Protein Acceptor

The prospect of achieving quantitative characterization of protein–ligand interactions originated in studies by Klotz [2], who demonstrated that the simplest model based on ligand interaction with *p* equivalent and independent sites on a protein (acceptor A) provided an adequate description of the binding of copper ions to bovine serum albumin. This extent of acceptor-ligand interaction was expressed in terms of a rectangular hyperbolic dependence of a binding function $r$ upon the molar concentration of free ligand, ${C}_{S}$. Specifically,

1 $$r = \frac{{\left( {\overline{C}_{S} - C_{S} } \right)}}{{\overline{C}_{A} }} = \frac{{pK_{AS} C_{S} }}{{1 + pK_{AS} C_{S} }}$$

where ${\overline{C} }_{S}$ and ${\overline{C} }_{A}$ denote the respective total molar concentrations of ligand and acceptor in the mixture being analysed; and where ${K}_{AS}$ is the intrinsic association constant for ligand interaction with $p$ equivalent and independent sites on the acceptor. Scatchard [1] facilitated the use of this rectangular hyperbolic dependence of the binding function upon free ligand concentration ${C}_{S}$ by its rearrangement to the form

2 $$\frac{r}{{C_{S} }} = pK_{AS} - K_{AS} r$$

which paved the way for evaluating $p$ and ${K}_{AS}$ from the slope and ordinate intercept of a linear dependence of $r/{C}_{S}$ upon $r$. This linear transform quickly found widespread application throughout the biological research community. Complications arose in situations where the Scatchard plot exhibited concave curvature, which was attributed either to non-equivalence or to negative cooperativity of the $p$ acceptor sites. No thought was given to the possibility that invalidity of assumed ligand univalence might be responsible for such curvature of a Scatchard plot.

## The Quantitative Expression for a Multivalent Ligand

The advent of quantitative affinity chromatography [3, 4] heightened the need for binding theory that encompassed ligand multivalence, because the macromolecular species now becomes an *f*-valent ligand that partitions between bound and free states. At this stage it is appropriate to present the corresponding expressions for interactions between an *f*-valent ligand, S, and a larger, *p*-valent macromolecular acceptor A ─ a situation pertinent to the characterization of immunochemical systems involving a multivalent protein antigen and its specific monoclonal antibody (IgG), or to size-exclusion chromatography studies in which the acceptor and all AS_i_ species comigrate [9, 10]. Indeed, use of the current approach was first illustrated with the characterization of the solution interaction between a bivalent lectin (concanavalin A) and Dextran T2000 by frontal size-exclusion chromatography on glyceryl-CPG 170 [8], where the chromatographic matrix merely provided a means for determining the free ligand concentration *C*_*S*_ in a solution with defined composition (${\overline{C} }_{A}, {\overline{C} }_{S}$). Although the problem of allowing for ligand multivalence was solved over 40 years ago, that development [5] seems to have escaped the notice of the biological community. The following derivation [11] may well gain greater acceptance because of closer adherence to the standard textbook approach introduced by Klotz [2] for univalent ligands.

### Derivation of the Scatchard Expression for a Multivalent Ligand

Consider the interaction of a ligand (S) bearing *f* equivalent and independent sites with affinity for the *p* equivalent and independent sites on acceptor (A). A ligand molecule is regarded as being bound to acceptor regardless of the number of its affinity sites involved in interactions with acceptor sites. The total molar concentration of ligand ($${\overline{C} }_{S})$$ then becomes the sum of the free ligand concentration ($${C}_{S})$$ and that of all complexes SA_*i*_ (1 $$\le i\le$$ *f*). In terms of stoichiometric binding constants ($${K}_{i}$$) considerations of mass conservation dictate that

3 $$\begin{aligned}\overline{C}_{S} &= C_{S} + pK_{1} C_{A} C_{S} + p^{2} K_{1} K_{2} C_{A}^{2} C_{S} \\ &\quad + \cdots + K_{1} K_{2} \ldots p^{f} K_{f} C_{A}^{f} C_{S}\end{aligned}$$

where $p{C}_{A}$ denotes the concentration of free acceptor sites. The assumed independence and equivalence of the *f* ligand sites allow the stoichiometric binding constants to be expressed as the product of the intrinsic (or site-binding) constant [2], ${K}_{AS}$, and the number of ways of forming the complex with stoichiometry SA_*i*_. Their elimination from Eq. (3) by means of the relationship [2]

4 $$K_{i} = [(f - 1 + 1)/i!]K_{AS}$$

leads to the expression

5a $$\begin{aligned} \overline{C}_{S} &= C_{S} + fpK_{AS} C_{A} C_{S} + [f\left( {f - 1} \right)/2!]p^{2} K_{AS}^{2} C_{A}^{2} C_{S} \\ & \quad + \cdots + p^{f} K_{AS}^{f} C_{A}^{f} C_{S} \\ \end{aligned}$$

or, on application of the binomial theorem,

5b $$\overline{C}_{S} = C_{S} ({1} + pK_{AS} C_{A} )^{f}$$

This equation is readily rearranged to the form

6 $$\left[ {(\overline{C}_{S} /C_{S} )^{1/f} - 1} \right] = pK_{AS} C_{A}$$

By analogous reasoning the total concentration of acceptor sites, $p{\overline{C} }_{A}$, is

7 $$\begin{aligned} p\overline{C}_{A} &= pC_{A} + fpK_{AS} C_{A} C_{S} + 2[f\left( {f - 1} \right)/2!]p^{2} K_{AS}^{2} C_{A}^{2} C_{S } \\ & \quad + \cdots + fp^{f} K_{AS}^{f} C_{A}^{f} C_{S} \\ & = pC_{A} + fpK_{AS} C_{A} C_{S} (1 + pK_{AS} C_{A} )^{f - 1} \\ \end{aligned}$$

Combination of the resultant expression for the concentration of free acceptor sites ($p{C}_{A}$) with Eq. (6) then gives

8 $$\begin{aligned}\left[ {(\overline{C}_{S} /C_{S} )^{1/f} - 1} \right] &= pK_{AS} \overline{C}_{A} C_{S}\\ &\quad- fpK_{AS}^{2} C_{A} C_{S} (1 + pK_{AS} C_{A} )^{f - 1}\end{aligned}$$

Removal of the terms in free acceptor concentration by substituting the left-hand side of Eq. (6) for the product $p{K}_{AS}{C}_{A}$ then yields the relationship

9 $$\frac{{\left( {\overline{C}_{S}^{1/f} - C_{S}^{1/f} } \right)}}{{C_{S}^{1/f} }} = pK_{AS} \overline{C}_{A} - fK_{AS} \overline{C}_{S}^{{\left( {f - 1} \right)/f}} \left( {\overline{C}_{S}^{1/f} - C_{S}^{1/f} } \right)$$

By defining the binding function for a multivalent ligand, ${r}_{f}$, as [8]

10 $$r_{f} = \frac{{\left[ {\overline{C}_{S}^{1/f} - C_{S}^{1/f} } \right]}}{{\overline{C}_{A} }}$$

Equation (9) becomes

11a $$r_{f} /C_{S}^{1/f} = pK_{AS} - fK_{AS} \overline{C}_{S}^{{\left( {f - 1} \right)/f}} r_{f}$$

or, in situations where the total concentration of acceptor sites ($p{\overline{C} }_{A}$) is also a parameter to be determined,

11b $$r_{f} \overline{C}_{A} /C_{S}^{1/f} = pK_{AS} \overline{C}_{A} - fK_{AS} \overline{C}_{S}^{{\left( {f - 1} \right)/f}} r_{f} \overline{C}_{A}$$

This Scatchard expression for a multivalent ligand was derived previously [8] from reacted-site probability theory. Researchers familiar with the traditional Scatchard analysis may query the presence of a term in total ligand concentration within the abscissa parameter. However, for a univalent ligand (*f* = 1) the term in question becomes unity because of the power (zero) to which ${\overline{C} }_{s}$ is raised. The traditional Scatchard expression, Eq. (2), is thus seen to reflect its generalized counterpart, Eq. (11a), for the situation in which the ligand is univalent.

### Early Days of Quantitative Allowance for Ligand Multivalence

As noted in Sect. 3, the initial breakthrough in allowance for ligand multivalence stemmed from the application of reacted-site probability [6] to obtain a complicated expression for the intrinsic binding constant in which ${K}_{AS}$ was the only parameter of unknown magnitude. For a system where the ligand S possesses *f* sites for equivalent and independent interaction with acceptor sites A, it follows from reacted-site probability theory [6] that the total and free concentrations of ligand are related by the expression

12 $$\overline{C}_{S} = \frac{{C_{S} }}{{(1 - P_{S} )^{f} }}$$

where ${P}_{S}$ denotes that probability that a site on the ligand has reacted with an acceptor site. The intrinsic binding constant for acceptor-ligand interaction, ${K}_{AS}$, may thus be written as

13 $$K_{AS} = \frac{{P_{S} }}{{\left( {1 - P_{S} } \right)\left( {p\overline{C}_{A} - fP_{S} \overline{C}_{S} } \right)}}$$

where the second term in the denominator is the free concentration of acceptor sites (p$${C}_{A})$$ expressed as the difference between the total concentration of acceptor sites and the concentration of bound acceptor sites. Combination of Eqs. (12) and (13) then gives [5]

14a $$K_{AS} = \frac{{[1 - (C_{S} /\overline{C}_{S} )^{1/f} ]}}{{(C_{S} /\overline{C}_{S} )^{1/f} [p\overline{C}_{A} - f\overline{C}_{S} \{ 1 - (C_{S} /\overline{C}_{S} )^{1/f} \} ]}}$$

or, on noting that $\{1-({C}_{S}/{\overline{C} }_{S}{)}^{1/f}\}$ = $\{({\overline{C} }_{S}/{C}_{S}{)}^{1/f}-1\}$/${{\overline{C} }_{S}}^{1/f}$

14b $$K_{AS} = \frac{{\{ (\overline{C}_{S} /C_{S} )^{1/f} - 1\} }}{{C_{S}^{1/f} [p\overline{C}_{A} - f\overline{C}_{S}^{{\left( {f - 1} \right)/f}} \{ {(}\overline{C}_{S} /C_{S} )^{1/f} - 1{\mathrm{\} }}]}}$$

which allows the determination of ${K}_{AS}$ as the sole parameter for a system with a defined total concentration of matrix sites, $p{\overline{C} }_{A}$. These complicated expressions for ${K}_{AS}$ provided the only means of quantifying a multivalent ligand interaction until the rearrangement [7] of Eq. (14a) as the linear transform

15 $$\begin{aligned}\frac{{\{ 1 - (C_{S} /\overline{C}_{S} )^{1/f} \} }}{{(C_{S} /\overline{C}_{S} )^{1/f} }} &= pK_{AS} \overline{C}_{A} \\ &\quad- fK_{AS} \overline{C}_{S} \{ 1 - (C_{S} /\overline{C}_{S} )^{1/f} \}\end{aligned}$$

or its equivalent formulation, Eq. (9), a relationship that was only developed [8] after the realization that Eq. (15) was a counterpart of the Scatchard expression for a multivalent ligand.

## Experimental Progress toward the Multivalent Scatchard Equation

As noted in relation to Eqs. (14(a) and (14b), the first breakthrough in allowance for the effects of ligand multivalence entailed their use to estimate the intrinsic binding constant (*K*_*AS*_) from [${\overline{C} }_{S}, {C}_{S}]$ measurements for defined concentrations of total acceptor sites ($p{\overline{C} }_{A})$. This initial approach is now illustrated with results from a partition equilibrium study deigned to quantify the interaction of a glycolytic enzyme with rabbit muscle myofibrils at physiological ionic strength.

### Partition Studies of the Biphasic Interaction Between Aldolase and Myofibrils

**Fig. 1:**
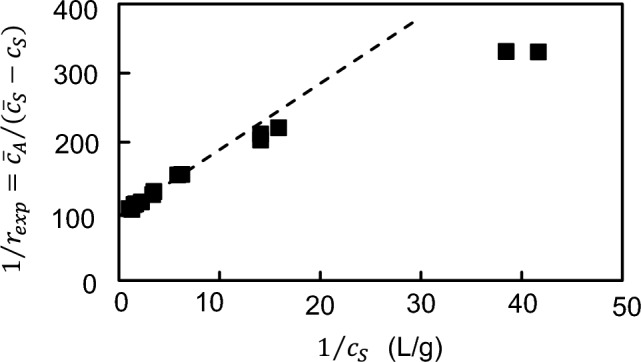
Determination of the binding capacity of muscle myofibrils for aldolase from a double-reciprocal plot of partition equilibrium data [12]. The dashed line denotes the limiting slope used to obtain the capacity (g enzyme/g myofibrils) as the reciprocal of the ordinate intercept

Results from partition studies [12] of the interaction between aldolase (S) and the myofibrillar matrix (A) in imidazole-chloride buffer (pH 6.8, *I* 0.158 M) (see Table 1 of [12]) were first used to define an experimental extent of matrix-bound enzyme, *r*_*exp*_ (g aldolase bound/g myofibrillar protein), from the difference between total ($${\overline{c} }_{S})$$ and free ($${c}_{S})$$ enzyme concentrations (g/L) for each total myofibrillar protein concentration $${\overline{c} }_{A}$$. The binding capacity of the myofibrillar matrix was then deduced from a double-reciprocal plot of the dependence of the extent of aldolase binding upon free aldolase concentration (Fig. 1). Because all accessible myofibrillar sites should be saturated at infinite free enzyme concentration (1/$${c}_{s}$$ → 0), the ordinate intercept was taken as the reciprocal of the maximal myofibrillar capacity: that value of 11.1 mg aldolase bound per g myofibrillar protein becomes 69.4 nmol/g based on a molecular weight of 160,000 for aldolase [13]. Multiplication of $${\overline{c} }_{A}$$ by this factor then gave the total myofibrillar site concentration ($$p{\overline{C} }_{A}$$) for each partition experiment; and hence rendered possible the use of Eq. (14a) to calculate the intrinsic binding constant as the only parameter of unknown magnitude for the tetrameric enzyme (*f* = 4). A *K*_*AS*_ of 410,000 (± 20,000) M^−1^ was signified by the mean of those measurements.

The same approach was also used [14] to rectify the interpretation of published Scatchard plots for the interactions of glycolytic enzymes with affinity sites on erythrocyte ghosts [15, 16].

### Scatchard Plots for Glycolytic Enzyme Interactions with Erythrocyte Ghosts

**Fig. 2:**
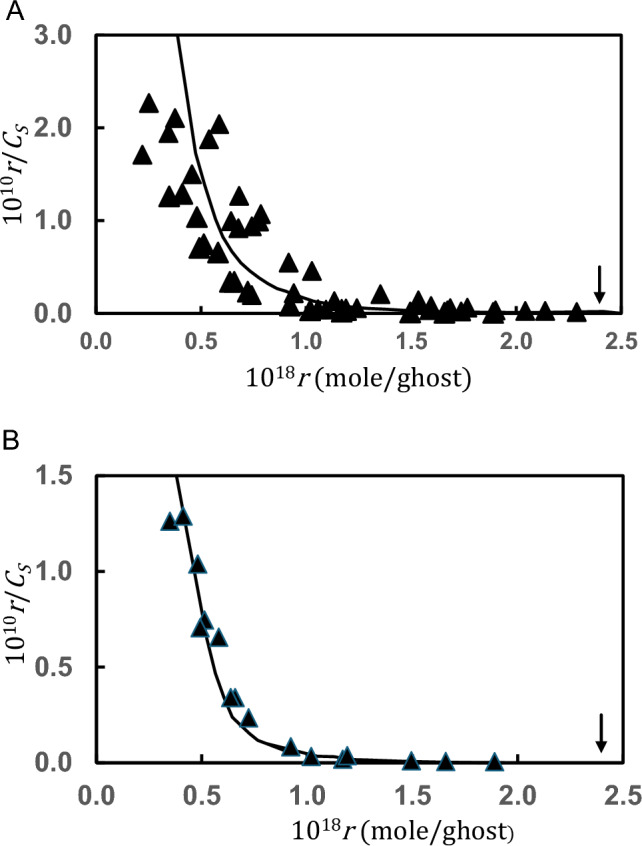
Conventional Scatchard plots of binding data for the interactions of human erythrocyte ghosts with aldolase (**A**) and glyeceraldehyde-3-phosphte dehydrogenase (**B**). Those data, taken from Fig. 2 of [15] and Fig. 1 of [16] respectively, are compared with the theoretical relationships predicted [14] by Eq. (14a) with respective association constants of 3.5 × 10^6^ M^─1^ and 1.0 × 10^6^ M^─1^ for the tetravalent enzymes with a single site on the erythrocyte ghosts. Vertical arrows denote the abscissa intercepts based on the band 3 content of the ghosts

Traditional Scatchard plots of results for the biphasic interactions of aldolase [15] and glyceraldehyde-3-phosphate dehydrogenase [16] with erythrocyte ghosts are presented in Figs. 2A, B respectively, where the vertical arrows denote the position of the abscissa intercept based on the band-3 protein content of erythrocyte ghosts. At the time the curvilinearity of these plots was taken to signify either heterogeneity or negative cooperativity of the band-3 protein sites to which glycolytic enzymes bind on the erythrocyte membrane surface.

Knowledge of ${\overline{C} }_{A}$ and ${C}_{S}$ made feasible the use of Eq. (14a) to calculate a value for ${K}_{AS}$ from each experimental [*C*_*S*_, *r*)] point, and hence to generate the predicted forms (——) of the traditional Scatchard plot based on the average ${K}_{AS}$ values and $f=4$[14].The findings presented in Figs. 2A, B clearly attest to the adequacy of their description in terms of equivalent and independent binding of a tetravalent protein to the band-3 protein located on the surface of erythrocyte ghosts ─ a conclusion consistent with the tetrameric quaternary structure of these glycolytic enzymes.

### Transition to a Linear Transform and the Multivalent Scatchard Plot

**Fig. 3:**
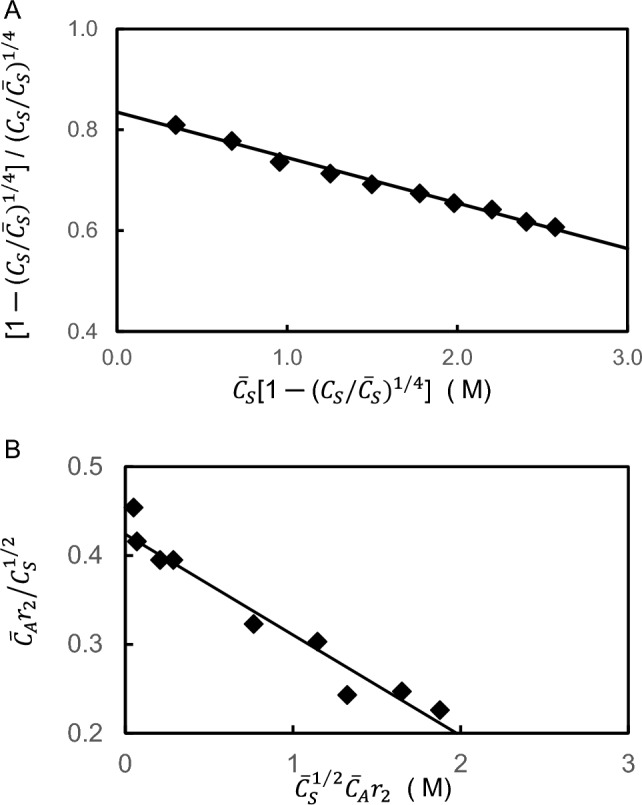
Allowance for multivalence of the partitioning species in quantitative affinity chromatography and size-exclusion chromatography. **A** An affinity chromatographic study of the interaction of tetravalent rabbit muscle lactate dehydrogenase with Sepharose Blue, where the results are potted in accordance with Eq. (15). (Data taken from Fig. 2 of [7].) **B** A size-exclusion chromatography study on glyceryl-coated porous glass beads of the interaction between bivalent concanavalin A and Dextran T2000, where the results are plotted in accordance with Eq. 11b). (Data taken from Fig. 1 of [8])

As noted in Sect. 3.1, the need to devise a means for determining ${\overline{C} }_{A}$ as a prerequisite for estimating ${K}_{AS}$ from Eq. (14a) can be avoided by using Eq. (15), its linear transform [7]. Values of ${\overline{C} }_{A}$ and ${K}_{AS}$ may therefore be obtained, for an assigned value of *f*, from the slope $(-\hspace{0.17em}f{K}_{AS}$) and ordinate intercept ${(K}_{AS}{\overline{C} }_{A}$) of a linear dependence of $[1-({C}_{S}/{\overline{C} }_{S}{)}^{1/f}]/({C}_{S}/{\overline{C} }_{S}{)}^{1/f}$ upon ${\overline{C} }_{S}[1-({C}_{S}/{\overline{C} }_{S}{)}^{1/f}]$. This procedure is illustrated in Fig. 3A, where data from a partition equilibrium study [7] of the interaction between rabbit muscle lactate dehydrogenase and Blue Sepharose CL-6B in imidazole-chloride buffer (pH 7.5 I 0.40 M) are analyzed by this means. Values of 2.3 (± 0.3) × 10^4^ M^−1^ and 37.2 (± 4.8) μM were obtained by linear last-squares analysis of those results [7].

Realization that substitution of a value of unity for *f* in Eq. (15) yielded the traditional Scatchard relationship, Eq. (2), quickly led to redefinition of a valence-specific binding function *r*_*f*_, Eq. (10), and a generalized Scatchard expression, Eq. (11a) or (11b). Use of this analysis was illustrated [8] by its application to the interaction between Dextran T2000 and concanavalin A, a ligand with two equivalent and independent binding sites for carbohydrate [17]. Frontal size-exclusion chromatography on a Glyceryl-CPG 170 column was used to determine the free lectin concentration (*C*_*S*_) in mixtures with defined composition [${\overline{c} }_{A},{\overline{C} }_{S})$] from the size of the slower-migrating boundary in the trailing elution profile in these experiments with comigration of acceptor and acceptor-ligand complexes [9, 10]. From the plot of those results in accordance with Eq. (11b) that is presented in Fig. 3B, an intrinsic association constant of 5,600 M^−1^ is obtained from the slope (−2*K*_*AS*_) for the polysaccharide interaction; and a value of 0.76 μM for the total concentration of acceptor sites (($p{K}_{AS}{\overline{C} }_{A}$) is signified by the ordinate intercept. However, because every glucose residue on the polysaccharide is a potential site for interaction with concanavalin A, this maximal capacity is an effective parameter governed by the size of the lectin, which obscures a far greater number of glucose residues than the one to which it has attached [18].

## Biological Systems Involving Multivalent Ligands

The applications designed to illustrate the advantages of employing the generalized Scatchard expression to allow for multivalence of the partitioning species have mainly employed model affinity chromatography systems. Attention is now turned to situations where the interactions of multivalent proteins with affinity sites on a particulate surface are of physiological significance.

**Fig. 4:**
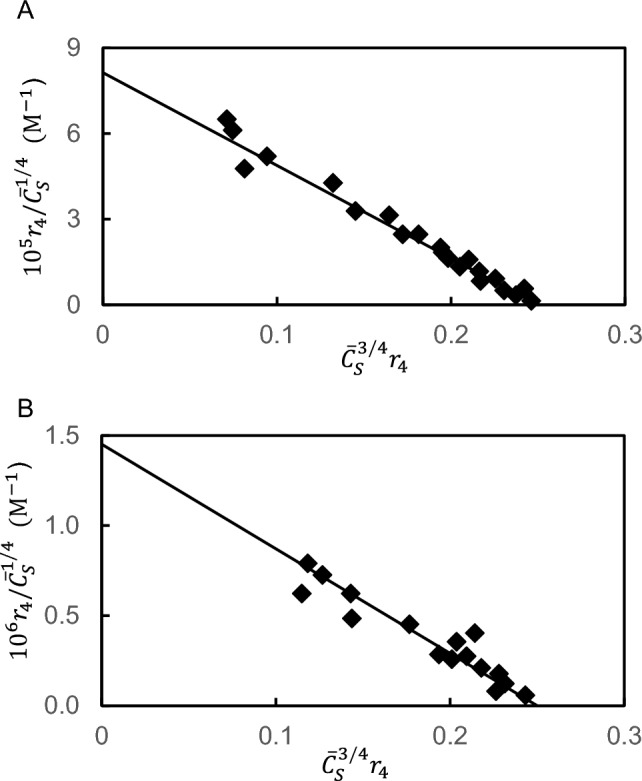
Characterization of the interactions of aldolase (**A**) and glyceraldehyde-3-phosphate dehydrogenase (**B**) with erythrocyte ghosts in 5 mM phosphate buffer (pH 7.4). Results are plotted according to Eq. (11a) with *f* = 4 for these tetravalent enzymes and the total concentration of ghost sites (${\overline{C} }_{A}$) based on the band 3 protein content. (Data taken from Fig. 1 of [19])

For that purpose, attention is first redirected to the interactions of glycolytic enzymes with erythrocyte ghosts that were introduced to highlight the need for a simpler means of quantitative characterization (Figs. 2A, B). Results from a subsequent study [19] of the same interactions are presented in Figs. 4A, B for aldolase and glyceraldehyde-3-phosphate dehydrogenase respectively, where allowance for tetravalence of the partitioning enzymes in accordance with Eq. (11a) has established definitively the equivalence and independence of their four sites with band-3 protein on the erythrocyte ghost surface. Equivalence and independence of glycolytic enzyme sites have also been observed in in their interactions with rabbit muscle myofibrils [20, 21].

### Competitive Radioimmunoassays: Amendment of the Müller Method

Although the Müller method [22, 23] is used to evaluate antibody-antigen binding constants, it is based on a value of unity for antigen valence. Allowance for multivalence of an antigen is readily incorporated into the binding expression for its reaction with antibody in radioimmunoassays. For the radioimmunoassay conducted in the absence of cold antigen the free (${C}_{{S}^{*}}$) and total (${\overline{C} }_{{S}^{*}})$ concentrations of labelled antigen are interrelated by the definition of the intrinsic binding constant (${K}_{{AS}^{*}}$) as [24, 25]

16 $$K_{{AS^{*} }} = \frac{{\left( {1 - \alpha_{0}^{1/f} } \right)}}{{\alpha_{0}^{1/f} \left[ {p\overline{C}_{A} - \left( {1 - \alpha_{0}^{1/f} } \right)\overline{C}_{{S^{*} }} } \right]}}$$

where ${\alpha}_{0}$ denotes the fraction of free labelled antigen, ${C}_{{S}^{*}}/{\overline{C} }_{{S}^{*}}$. Rearrangement of Eq. (16) as

17 $$pK_{{AS^{*} }} \overline{C}_{A} = \frac{{\left( {1 - \alpha_{0}^{1/f} } \right)}}{{\alpha_{0}^{1/f} }} + K_{{AS^{*} }} \left( {1 - \alpha_{0} } \right)\overline{C}_{{S^{*} }}$$

yields an expression for the product $p{K}_{{AS}^{*}}{\overline{C} }_{A}$. Provided that the antibody exhibits the same affinity for hot and cold antigen $({K}_{{AS}^{*}}={K}_{AS})$, the corresponding expression for experiments with mixtures of S and S* is

18 $$pK_{{AS^{*} }} \overline{C}_{A} = \frac{{\left( {1 - \alpha_{S}^{1/f} } \right)}}{{\alpha_{S}^{1/f} }} + K_{{AS^{*} }} \left( {1 - \alpha_{S} } \right)\left( {\overline{C}_{{S^{*} }} + \overline{C}_{S} } \right)$$

where ${\alpha}_{S}={C}_{{S}^{*}}/{\overline{C} }_{{S}^{*}}$ is the free fraction of antigen (labelled and cold) in the presence of total antigen concentration $({\overline{C} }_{{S}^{*}}+{\overline{C} }_{S})$. Elimination of $p{K}_{{AS}^{*}}{\overline{C} }_{A}$ between Eqs. (17, 18) then gives [24, 25]

19 $$\begin{aligned}\frac{{\left( {1 - \alpha_{o}^{1/f} } \right)}}{{\alpha_{0}^{1/f} }} - \frac{{\left( {1 - \alpha_{S}^{1/f} } \right)}}{{\alpha_{s}^{1/f} }} &= fK_{{AS^{*} }} \left( {1 - \alpha_{S}^{1/f} } \right)\left( {\overline{C}_{{S^{*} }} + \overline{C}_{S} } \right)\\ &\quad- \left( {1 - \alpha_{0}^{1/f} } \right)\overline{C}_{{S^{*} }}\end{aligned}$$

Upon setting *f* = 1, ($1-{\alpha}_{0})=0.5$ and ($1-{\alpha}_{S})=0.25,$ Eq. (19) simplifies to the Müller expression [22, 23]. The present approach is thus superior to the Müller method in two respects: not only does it allow the quantitative characterization of multivalent antigens but also the analysis of all data for a system involving a univalent antigen (hapten).

In the presence of a total concentration ${\overline{C} }_{i}$ of an *f*-valent competitive inhibitor of radiolabelled antigen binding, the distribution of labelled ligand is related to the intrinsic inhibitor constant, ${K}_{AI}$, by the expressions [24, 25]

20 $$K_{AI} = \frac{{\left( {1 - \beta } \right)f\overline{C}_{I} }}{{K_{{AS^{*} }} \alpha_{i}^{1/f} \left[ {\left( {1 - \alpha_{i}^{1/f} } \right)\beta f\overline{C}_{I} } \right]}}$$

21 $$\begin{aligned}\left( {1 - \beta } \right)f\overline{C}_{I} &= f\overline{C}_{{S^{*} }} \left( {\alpha_{i}^{1/f} - \alpha_{0}^{1/f} } \right)\\ &\quad+ K_{{AS^{*} }} \left[ {\frac{{\left( {1 - \alpha_{0}^{1/f} } \right)}}{{\alpha_{0}^{1/f} }} - \frac{{\left( {1 - \alpha_{i}^{1/f} } \right)}}{{\alpha_{i}^{1/f} }}} \right]\end{aligned}$$

where ${\alpha}_{0}$ and ${\alpha}_{i}$ denote the respective fractions of free *f*-valent labelled antigen in the absence and presence of inhibitor.

**Fig. 5:**
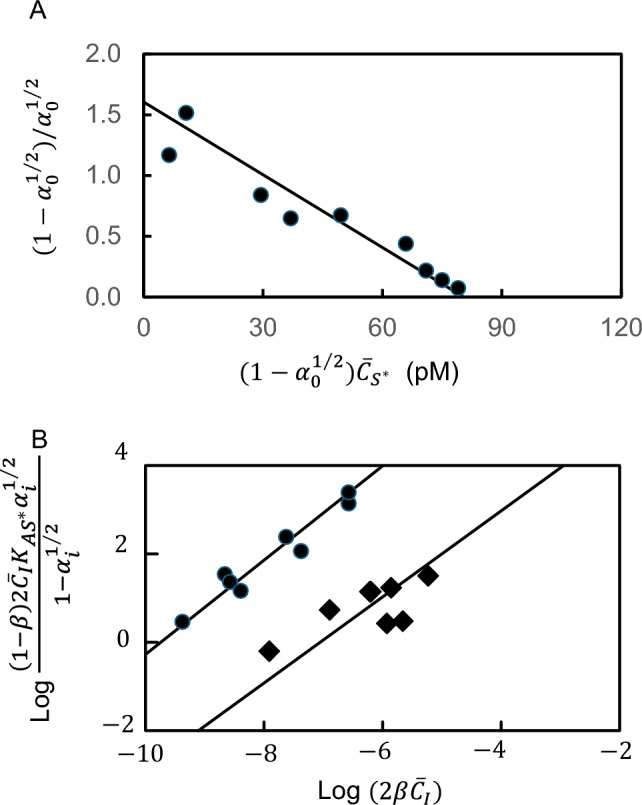
Allowance for multivalence of the antigen in the characterization of interactions by competitive radioimmunoassay. **A** Multivalent Scatchard plot to evaluate ${K}_{A{S}^{*}}$ for the interaction of radiolabelled fibrinogen, a bivalent antigen, with an elicited antibody. **B** Application of Eqs. (20) and (21) to determine ${K}_{AI}$ for the competitive inhibition by native fibrinogen (●) and its disulfide knot fragment (◆). (Data taken from [25])

Analyses of radioimmunoassay data for the binding of [^125^I]fibrinogen (S*) and two fibrinopeptides (I) to an anti-fibrinogen antibody [25] are summarized in Fig. 5. The evaluation of ${K}_{{AS}^{*}}$ by multivalent Scatchard analysis [Eq. (16)] of results for mixtures of antibody (A) and radiolabelled fibrinogen is presented in Fig. 5A, whereas the logarithmic form of Eq. (20) is used in Fig. 5B to determine ${K}_{AI}$ from the ordinate intercepts of data for the competitive interactions of fibrinogen and the disulfide knot derived therefrom. Consideration of native fibrinogen to be a competitive inhibitor of [^125^I]fibrinogen binding has led to a value of 8.1 ($\pm \hspace{0.17em}$3.0) $\times \hspace{0.17em}$10^9^ M^−1^ for ${K}_{AI}$ that essentially duplicates the estimate of 1.0 ($\pm \hspace{0.17em}$0.1) $\times \hspace{0.17em}$10^10^ M^−1^ for ${K}_{{AS}^{*}}$ that emanates from Fig. 5A. The observation of a significant but weaker interaction (${K}_{AI}=2.1\times {10}^{7}$ M^−1^) for the bivalent disulfide knot fragment with anti-fibrinogen can be attributed to the fact that the antibody was an elicited response to the whole fibrinogen molecule.

### Linearity of Traditional Scatchard Plots for Multivalent Ligands

**Fig. 6:**
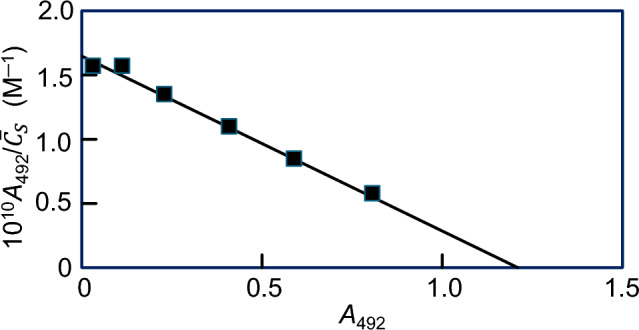
Traditional Scatchard plot of results obtained by an ELISA for ferritin: the total antigen concentration has been substituted for its free counterpart because of their essential identity. (Data taken from Fig. 4 of [26])

Traditional Scatchard plots can still be linear despite multivalence of the partitioning protein, as noted in the original detailed development of quantitative affinity chromatography theory [4]. This situation is encountered [26] in the FERRIZYME test system, a solid-phase immunoassay based on the sandwich principle. A bead coated with anti-ferritin is first incubated with the antigen before reaction with a solution of anti-ferritin that has horse radish peroxidase covalently attached: the enzymatic activity of the bead towards an *o*-phenylenediamine/H_2_O_2_ solution is then assayed by colorimetry. The intriguing feature of this assay system is the rectangular hyperbolic dependence of the calibration plot (*A*_492_ vs *C*_*S*_) provided by the manufacturer. This feature of the assay is confirmed by linearity of the corresponding Scatchard plot (Fig. 6) for defining the concentration of ferritin, an antigen with very great potential for multivalence because of the 24 identical subunits comprising this iron-storage protein [27].

The mathematical explanation of that inference came to light much later in a quantitative BIAcore study of the interaction of concanavalin A with the carboxymethylated dextran layer on the biosensor chip [28]. In situations where the concentration of bound analyte (S) is small relative to its free concentration, the total analyte concentration can be written as

22 $$\overline{C}_{S} = C_{S} + \left( {\overline{C}_{S} - C_{S} } \right) = C_{S} \left( {1 + \delta } \right)$$

where $\delta = \left( {\overline{C}_{S} - C_{S} } \right)/C_{S}$ is much less that unity. From the binomial theorem it then follows that

23 $$\overline{C}_{S}^{1/f} \approx C_{S}^{1/f} \left( {1 + {\delta \mathord{\left/ {\vphantom {\delta f}} \right. \kern-0pt} f}} \right)$$

in which case the multivalent Scatchard expression becomes

24 $$\frac{{\left( {\overline{C}_{S} - C_{S} } \right)}}{{\overline{C}_{S} }} = pK_{AS} \overline{C}_{A} - fK_{AS} \left( {\overline{C}_{S} - C_{S} } \right)$$

The plot of bound/free analyte upon bound analyte is thus a conventional univalent Scatchard plot, except that the slope defines $f{K}_{AS}$ ─ the intuitive prediction two decades earlier [4].

In similar fashion, the sensitivity of binding response in many solid-phase immunoassays such as ELISA also suffices [29, 30] to allow adoption of the approximate expression for ${\overline{C} }_{S}^{1/f}$, Eq. (23), and hence account to be taken for IgG antibody bivalence in accordance with Eq. (24).

## The Rectangular Hyperbolic Expression

The final question to be addressed in this review concerns a search for the rectangular relationship of which Eqs. (11a, 11b) are the linear transforms ─ an unusual situation in that a linear transform is usually sought to facilitate evaluation of the two basic parameters,${K}_{AS}, {\overline{C} }_{A}$, describing the rectangular hyperbolic dependence. Here the problem is reversed: Eqs. (11a, 11b) were obtained without recourse to the rectangular hyperbolic relationship of which they are linear transforms. Rearrangement of Eq. (11a) yields the expression [31]

25 $$r_{f} = \frac{{K_{AS} C_{S}^{1/f} }}{{1 + fK_{AS} \overline{C}_{S}^{{\left( {f - 1} \right)/f}} C_{S}^{1/f} }}$$

which, on multiplication throughout by $f{\overline{C} }_{A}^{(f-1)/f}$ becomes

26 $$fr_{f} C_{S}^{{\left( {f - 1} \right)/f}} = \frac{{fC_{S}^{{\left( {f - 1} \right)/f}} K_{AS} C_{S}^{1/f} }}{{1 + fK_{AS} \overline{C}_{S}^{(f - 1/f} C_{S}^{1/f} }}$$

Equation (26) describes a rectangular hyperbolic dependence of $f{{r}_{f}C}_{S}^{(f-1)/f}$ upon ${\overline{C} }_{S}^{(f-1/f}{C}_{S}^{1/f}$. Systems for which the total concentration of acceptor sites $p{\overline{C} }_{A}$ is also to be evaluated as a curve-fitting parameter are accommodated by multiplying Eq. (26) throughout by ${\overline{C} }_{A}$ to yield an expression in ${r}_{f}{\overline{C} }_{A}$, as in Eq. (11b), which is simply $\left({\overline{C} }_{S}^{1/f}-{C}_{S}^{1/f}\right)$. With that modification Eq. (26) becomes

27 $$f\overline{C}_{S}^{{\left( {f - 1} \right)/f}} \left( {\overline{C}_{S}^{1/f} - C_{S}^{1/f} } \right) = \frac{{fK_{AS} \overline{C}_{S}^{{\left( {f - 1} \right)/f}} C_{S}^{1/f} }}{{1 + fK_{AS} \overline{C}_{S}^{{\left( {f - 1} \right)/f}} C_{S}^{1/f} }}$$

**Fig. 7:**
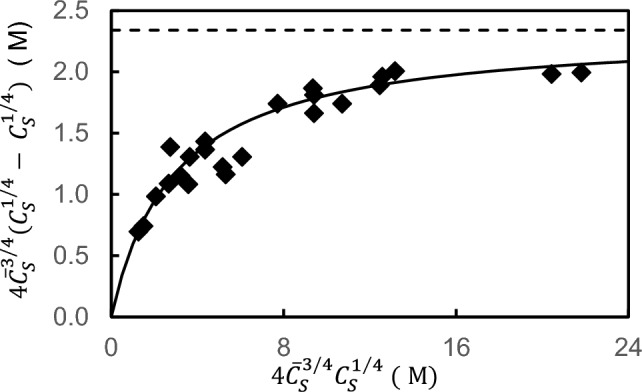
Demonstration of the rectangular hyperbolic relationship of which the generalized Scatchard expression is the linear transform. Use of Eq. (27) to characterize the interaction of aldolase with muscle myofibrils [21]: the solid line is the theoretical relationship for a system with *K*_*AS*_ = 3.4 × 10^5^ M^─1^ and a myofibrillar site concentration ($p{\overline{C} }_{A})$ of 2.34 μM (dashed line). (Data taken from Fig. 3 of [21])

The use of this expression to obtain $p{\overline{C} }_{A}$ as well as ${K}_{AS}$ from results for the interaction of aldolase with muscle myofibrils [21] is shown in Fig. 7.

## Concluding Remarks

Forty years have elapsed since these methods of allowance for the consequences of ligand (or analyte) multivalence in binding studies were developed. Their failure to attract interest within the biological research community is surprising in view of the importance of protein interactions in physiological, pharmacological and immunological control. Reluctance to accept a Scatchard expression for multivalent ligands based on reacted-site probability theory [6] does not appear to have been an issue, because a subsequent derivation [11] with closer adherence to the standard Klotz approach [2] had no impact. Admittedly, a contributing factor to its neglect has been the massive switch to molecular genetics as the way to solve all biological malfunctions. Another cause for disinterest may well have been the lack of computer programs written specifically for their application. In that regard programs for linear lest-squares analysis abound, as do those for nonlinear regression analysis to characterize a rectangular hyperbolic dependence. All that is required of a researcher is a preparedness to enter appropriate values of the independent and dependent variables in these widely available computer programs.

This review has summarized the status of the field for any researcher willing to take on the challenge of quantitatively characterizing macromolecular interactions involving the existence of a multivalent ligand in bound and free states.

## Data Availability

No datasets were generated or analysed during the current study.
